# Bioelectrical Impedance Analysis as a Non-Invasive Approach to Estimate *In Vivo* Body Composition in Rabbit Does Across Physiological Stages

**DOI:** 10.3390/ani15243611

**Published:** 2025-12-15

**Authors:** Nuria Nicodemus, Nelly Pereda, Joaquín Fuentespila, Pedro L. Lorenzo, Pilar G. Rebollar

**Affiliations:** 1 Departamento de Producción Agraria, ETSI Agronómica, Alimentaria y de Biosistemas, Universidad Politécnica de Madrid, 28040 Madrid, Spain; nluciapereda@gmail.com (N.P.); pilar.grebollar@upm.es (P.G.R.); 2 Departamento de Economía Agraria, Estadística y Gestión de Empresas, ETSI Agronómica, Alimentaria y de Biosistemas, Universidad Politécnica de Madrid, 28040 Madrid, Spain; joaquin.fuentespila@upm.es; 3 Departamento de Fisiología, Facultad de Veterinaria, Universidad Complutense de Madrid, Ciudad Universitaria, 28040 Madrid, Spain; plorenzo@vet.ucm.es

**Keywords:** bioelectrical impedance analysis, body composition, rabbit does, reproductive physiology, prediction equations, validation

## Abstract

This study aimed to develop and validate bioelectrical impedance analysis (BIA)-based prediction equations for estimating the *in vivo* body composition of reproductive rabbit does across different physiological stages. A total of 87 New Zealand × Californian rabbit does were used to generate calibration models, and 25 additional rabbit does served for independent validation. Animals were categorized according to reproductive status (nulliparous, pregnant-lactating, pregnant-non-lactating, non-pregnant-lactating, and non-pregnant-non-lactating). BIA measurements were obtained using a Quantum II analyzer, and chemical composition was determined by proximate analysis. Multiple linear regression models were developed, and equations were validated through relative mean prediction error (RMPE). Significant effects of physiological status were observed on body composition: pregnant-lactating does showed the highest water content, while non-pregnant-non-lactating females exhibited the greatest protein and fat concentrations. Fat and energy contents decreased markedly (−24% and −32%, respectively) during lactation, indicating intense metabolic mobilization. Regression models revealed strong correlations between impedance parameters and chemical composition. Validation confirmed high predictive accuracy (RMPE 15–25%), with crude protein slightly underestimated (3–4%). These findings confirm that BIA provides a reliable, non-destructive alternative to comparative slaughter for assessing body composition in breeding rabbit does throughout the reproductive cycle.

## Simple Summary

The assessment of temporal changes in the body chemical composition of reproductive does across successive reproductive cycles is crucial for research, technical, and commercial applications, as it contributes to optimizing reproductive performance and lifespan. Furthermore, the implementation of non-invasive techniques is desirable to ensure animal welfare. The present study demonstrates that bioelectrical impedance analysis (BIA) provides an accurate and non-invasive approach for evaluating the dynamic changes in the chemical composition of female rabbits throughout their productive lifespan, eliminating the need for slaughter.

## 1. Introduction

The body condition of female rabbits has been consistently associated with their reproductive performance [1,2] and longevity [3,4]. Recent evidence indicates that obesity represents one of the major risk factors for culling and mortality in rabbit does, particularly in nulliparous and multiparous females, as it predisposes them to metabolic disorders such as hepatic lipidosis and requires specific management in breeding stock [5]. Traditionally, comparative slaughter has been regarded as the reference method for determining whole-body chemical composition in rabbits [6,7,8,9]. However, this approach is restricted to experimental settings and does not allow for longitudinal monitoring of body composition across multiple reproductive cycles.

Researchers have attempted to develop *in vivo* approaches to predict body composition using indirect methods such as near-infrared spectroscopy (NIRS) [10,11], deuterium oxide (D_2_O) dilution [12], magnetic resonance imaging (MRI) [13,14], and X-ray computerized tomography (CT) [15,16]. Although these techniques are of considerable interest, they are expensive, particularly for this domestic species, and technically demanding, which limits their feasibility as practical alternatives to comparative slaughter.

For this reason, considerable research efforts in recent years have focused on developing *in vivo* methodologies that allow for an affordable assessment of body composition in rabbit does. Among these techniques, the following can be highlighted: total body electrical conductivity (TOBEC; [17]), ultrasonographic assessment of perirenal fat thickness [18], and body condition scoring (BCS; [1]). Nevertheless, the accuracy of TOBEC in rabbit does has been questioned, as it tends to yield unreliable estimates of protein and ash content [17]. Although they are not exactly refining methods, their use reduces the number of slaughtered animals, so they would be considered in line with the use of alternative methods that pursue any of the 3Rs [19].

Bioelectrical impedance analysis (BIA) has emerged as a promising alternative. The principles of BIA are based on the body’s opposition to the passage of an alternating electric current, which comprises two components: reactance (*Xc*), originating from cell membranes, and resistance (*Rs*), associated with intra and extracellular fluids [20]. The total impedance (*Z*) combines both components and is calculated as $Z=\sqrt{Rs^{2}\;+\;Xc^{2}}$. Assuming constant body geometry and applying a standardized alternating current, animals with a greater proportion of adipose tissue exhibit higher impedance values, due to the low electrical conductivity of fat [21].

BIA has been extensively applied to estimate body composition in humans [22,23], pigs [21,24,25,26], lambs [27,28], beef cattle [29,30], steers [31], fish [32], and goats [33]. More recently, BIA has been validated in growing rabbits to predict carcass composition and nutrient retention [34,35], as well as in broiler chickens [36]. These studies highlight its main advantages, namely high accuracy, repeatability, and non-invasiveness.

In addition, BIA has been employed extensively in studies on the reproductive and nutritional physiology of rabbit does carried out by our research group. For instance, in previous work, we have applied our BIA-based prediction equations to assess metabolic status, monitoring indices such as blood leptin and non-esterified fatty acid (NEFA) levels as indicators of body reserve mobilization [37], and to explore how body reserves relate to fertility performance, finding that higher body protein and fat contents were associated with improved conception rates and litter outcomes [3,38,39]. We also employed these equations to evaluate the effects of dietary regimens [40,41,42,43,44] and weaning and reproductive management strategies [45] on the rabbit does’ body composition. In all these cases, BIA-derived composition estimates provided a minimally invasive approach to monitor changes in fat and energy reserves, thereby linking the nutritional and metabolic status of the rabbit does to key outcomes such as endocrine profiles, reproductive performance, and long-term body condition stability. However, although the prediction equations used in those studies had been previously developed and validated by our group as part of a doctoral dissertation in 2010 [46], to date, neither the equations nor the validation procedures had been published in peer-reviewed scientific journals for this category of animals. Consequently, the scientific community still lacks access to reliable BIA models for estimating the body composition of reproductive does *in vivo*.

Therefore, the objective of the present work is to fill this gap, showing these BIA-based prediction equations and their validation procedures to estimate the *in vivo* body composition of rabbit does at different physiological stages throughout the reproductive cycle. By making these equations accessible to the scientific community, this work provides researchers and practitioners with a new tool to assess body composition in reproductive does, analogous to the models already established for young growing rabbits and broiler chickens. This will allow others to utilize BIA in reproductive does, something that has not been possible until now due to the absence of published equations.

## 2. Materials and Methods

This work was carried out in 2008–2009, in the context of agricultural research, and according to the regulations in force [47], non-experimental agricultural practices and veterinary clinics are excluded from the scope of this directive. Therefore, animals had to be kept under conditions similar to those of animals in commercial farms, and their housing complied with the standards laid out in the Spanish legislation [48], which incorporates the European Directive on the protection of animals kept for farming purposes into Spanish law. In addition, animals were handled according to the principles for the care of animals in experimentation [47,49,50] and favorably assessed retrospectively by the Ethics Committee of the Polytechnic University of Madrid (code: CE251212).

### 2.1. Animals and Housing

A total of 87 New Zealand × Californian rabbit does, weighing between 3002 and 5736 g, were used as the calibration group (CG) to develop regression equations for the *in vivo* estimation of body composition. All rabbit does were artificially inseminated 11 days after parturition, and their litters were weaned at 35 days of age.

Animals were allocated to five groups according to their physiological status: nulliparous (16–19 weeks old; NUL; *n* = 15), pregnant (21 days of gestation) and lactating (32 days of lactation; PL; *n* = 18), pregnant (23–28 days of gestation) and non-lactating (PNL; *n* = 18), non-pregnant and lactating (11 days of lactation, at insemination; NPL; *n* = 18), and non-pregnant and non-lactating (NPNL; *n* = 18). An additional set of 25 females (5 per physiological category), weighing between 2837 and 5014 g, was used as the validation group (VG) to assess the predictive accuracy of the equations generated from the CG. Parity order within each reproductive category ranged from 0 to 10 kindlings. All animals had *ad libitum* access to water until slaughter.

A commercial diet (Cunimax-A, Cargill SA, Spain; 18.5 MJ GE/kg DM, 188 g CP/kg DM, and 388 g NDF/kg DM) was provided *ad libitum* during late pregnancy (from day 28 onwards) and throughout lactation, whereas feed intake was restricted to 150 g/day from weaning until day 28 of gestation. Diet contained a vitamin and mineral premix (provided per kg of diet: vitamin A, 10,000 I.U.; vitamin D3, 900 I.U.; riboflavin, 3 mg; calcium d-pantothenate, 10 mg; nicotinic acid, 25 mg; menadione, 1 mg; alpha-tocopherol, 35 mg; thiamine, 1; pyridoxine, 1.5 mg; biotin, 0.05 mg; folic acid, 1.5 mg; cianocobalamin, 0.012 mg; manganese, 15 mg; zinc, 50 mg; iodine, 0.8; iron, 40 mg; copper, 8 mg; cobalt, 0.30 mg; selenium, 0.05 mg; robenidine, 50 mg) and 100 mg zinc-bacitracin/kg (APSA, Reus, Spain).

Rabbit does were individually housed at the facilities of the Universidad Politécnica de Madrid in flat-deck cages measuring 700 × 500 × 320 mm (length, width and height, respectively), under controlled environmental conditions (ambient temperature between 16 and 24 °C, forced ventilation, and a light/dark photoperiod of 16:8 h). Light was switched on at 07:30 h a.m.

### 2.2. Bioelectrical Impedance Analysis Measurements

Bioelectrical impedance was measured using a four-terminal body composition analyzer (Quantum II, Model BIA-101, RJL Systems, Detroit, MI, USA). Prior to each measurement session, the device was calibrated using a standard 500 Ω resistor to verify the accuracy of the system. A constant alternating current of 800 µA at 50 kHz was delivered through the black transmitter leads (two distal electrodes), while resistance (*Rs*, Ω) and reactance (*Xc*, Ω) were recorded via the red detector leads (two proximal electrodes).

**Figure 1:**
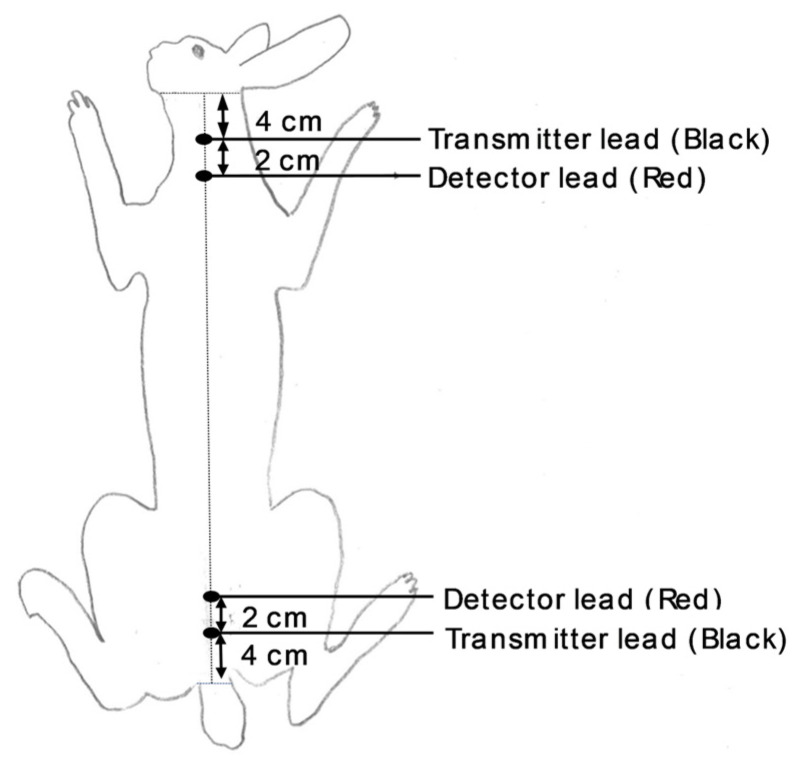
Electrode locations in live doe rabbit body.

Rabbit does were neither anesthetized nor shaved during the procedure. Animals were positioned on a flat, non-conductive, non-slip board that provided a secure surface. Standard stainless-steel hypodermic needles (21 G × 1½″, 0.8 × 40 mm) were used as electrodes and inserted subcutaneously through the skin, which did not cause any pain, allowing the rabbit does to stay calm throughout the measurements. Bleeding rarely occurred with the insertion of the needles, but if any superficial capillaries were ever reached, the skin was disinfected with an antiseptic solution. Electrodes were positioned along the dorsal midline: for the distal transmitter pair, one electrode was inserted 4 cm caudal to the base of the ears (scapular region) and the other 4 cm cranial to the base of the tail (rump region). The proximal detector pair was placed 2 cm caudal (scapular region) and 2 cm cranial (rump region) to the respective transmitter electrodes (Figure 1).

The distance between detector electrodes (D, cm) and the dorsal length (L, cm) from the base of the ears to the base of the tail were measured using a flexible steel tape. Live weight (LW, g) and parity order (PO) were also recorded for each female. Bioelectrical impedance measurements were taken twice per animal in calibration and validation groups (at 30 min- intervals) between 09:00 and 11:00 h to assess measurement repeatability. Because feed and water intake in rabbits at this time is minimal [51], nutritional and hydration status were not standardized before taking BIA measurements. Furthermore, the measurements were taken under conditions similar to those we might find on commercial farms. The approximate time required to measure one animal (including the weighing of the animal and electrode insertion and removal) was around two minutes.

### 2.3. Slaughtering and Processing of the Samples

Following BIA measurements, the staff with required accreditations euthanized animals using an intravenous administration of sodium pentobarbital (Dolethal^®^, Vetoquinol, Madrid, Spain) at a dose of 120 mg/kg body weight, injected into the marginal ear vein (2–2.5 mL per doe, depending on body weight). Barbiturates were administered at low doses for sedation, and once adequate sedation was achieved, the euthanasia dose was subsequently administered. After euthanasia, carcasses were stored at −20 °C until processing.

Before grinding, each carcass was thawed slowly for 24 h at 4 °C and subsequently chopped into small pieces. Entire animals, including skin, hair, and major organ systems, were then homogenized using an industrial meat grinder (Cruells, C-15 EN 60742). A representative portion of the homogenate was collected from each rabbit. One aliquot was immediately analyzed for water content, while the remaining sample was refrozen at −20 °C. Samples were later freeze-dried for 72 h and milled through a 1 mm screen prior to chemical analyses.

### 2.4. Analytical Methods

Dry matter (DM) content of the ground material was determined by mixing 5 g of sample with 20 g of sea sand and 5 mL of ethanol, followed by drying at 103 °C for 24 h, according to the ISO 1442 method [52]. Chemical analyses were performed according to AOAC procedures [53]: DM (oven-drying method, 934.01), ash (muffle furnace incineration, 923.03), ether extract (Soxhlet extraction following 3 N HCl acid hydrolysis, 920.39), and crude protein (CP), using the Dumas combustion method (968.06) with an FP-528 analyzer (LECO, St. Joseph, MI, USA). Gross energy (GE) was determined by isoperibol bomb calorimetry (Model 1356, Parr Instrument Company, Moline, IL, USA).

### 2.5. Statistical Analysis

The effects of physiological state on body composition of rabbit does were analyzed using a completely randomized design, with parity order (PO) included as a linear covariate and physiological state as the main fixed effect. Data were analyzed using the GLM procedure of SAS [54]. Results are presented as least-squares means (LSMeans), and pairwise comparisons among physiological states were performed using the *t*-test.

Repeatability (S_R_), representing the intra-series variability of BIA measurements within individual rabbit does, was estimated using the VARCOMP procedure of SAS. It was calculated as S_R_ = √(Se^2^), where *Se* denotes the expected variance of error. The coefficient of variation in repeatability (CV_R_) was expressed as the ratio between S_R_ and the mean BIA value, multiplied by 100.

Pearson correlation coefficients between BIA variables and carcass chemical composition were computed using the CORR procedure.

To identify the regression models that best explained the variation in the dependent variables, the RSQUARE option of the REG procedure was applied using data from the calibration group (CG). Dependent variables included water (expressed as % and g), crude protein (CP), ash, fat (expressed as % DM and g), and gross energy (kJ/100 g DM and MJ). Independent variables considered as potential predictors were physiological state (NUL, PL, PNL, NPL, NPNL), PO, PO^2^, live weight (LW, LW^2^), distance between detector electrodes (D, D^2^), dorsal length (L, L^2^), resistance (*Rs*, *Rs*^2^), reactance (*Xc*, *Xc*^2^), impedance (*Z, Z*^2^), and derived volume indices vol_1_ (D^2^/*Rs*) and vol_2_ (D^2^/*Z*).

Model selection was based on Mallows’ *Cp* statistic [55], ensuring values ≤ *p* + 1 (where *p* is the number of independent variables) to avoid bias due to omission of relevant predictors. Among models meeting this criterion, the optimal model was selected according to the minimum values of the following criteria: SP Statistic [56], Final Prediction Error (JP) [56,57], Amemiya’s Prediction Criterion (PC) [57,58], and Akaike’s Information Criterion (AIC) [59].

Once the most appropriate predictors were identified, parameter estimation for the multiple linear regression (MLR) models was performed using the REG procedure. Validation of the regression equations was conducted using independent data from the validation group (VG).

Prediction accuracy was evaluated using the Mean Prediction Error (MPE), calculated as the square root of the mean squared difference between the observed (chemically determined) and predicted values of each body composition parameter. The Relative Mean Prediction Error (RMPE, %) was expressed as the ratio between MPE and the mean observed value of the corresponding parameter. Differences between observed and predicted values derived from MLR equations in the validation group were assessed using paired *t*-tests.

## 3. Results

### 3.1. Chemical Composition of Rabbit Does

The chemical composition of the rabbit does used for the development and validation of the prediction equations is presented in Table 1. The average chemical composition of the calibration group was comparable to that of the validation group, indicating a consistent baseline between both datasets.

Table 2 presents the effect of physiological status on body composition in rabbit does from the calibration group. The live weight of pregnant-lactating (PL) and pregnant-non-lactating (PNL) females was 7.5% and 19.5% higher (*p* < 0.001), respectively, than that of non-pregnant-lactating (NPL) and nulliparous (NUL) does. Non-pregnant, non-lactating (NPNL) females exhibited intermediate body weights.

When chemical composition was expressed on a percentage basis, water content was significantly higher (*p* < 0.001) in PL does compared with the other physiological groups, with the lowest values recorded in NPNL and NUL does. NPL and PNL does showed intermediate values. The highest crude protein content was observed in NPNL rabbit does (*p* < 0.001) compared with all other physiological states. Conversely, PL does showed a marked reduction (*p* < 0.001) in fat (−24%) and energy (−32%) contents relative to the mean values of the remaining groups. No significant differences were detected in ash content, which averaged 3.14% across all groups.

The variations in chemical composition among physiological states, when expressed in absolute values (g), followed the same trend as those observed when expressed on a percentage basis, as shown in Table 2.

### 3.2. Impedance Measurements and Repeatability

Mean (±SD) values for resistance (*Rs*), reactance (*Xc*), and inter-electrode distance (D) in calibration group rabbit does were 106 ± 20.7 Ω, 25.2 ± 7.53 Ω, and 20.2 ± 2.06 cm, respectively (Table 3).

In Table 4 the values of repeatability (S_R_, Ω) and coefficient of variation in repeatability (CV_R_, %) of *Rs* and *Xc* are shown. The within-animal standard deviation of repeated measurements (repeatability, S_R_) was greater for resistance than for reactance. Nevertheless, when expressed as the coefficient of variation in repeatability (CV_R_), resistance exhibited values approximately eleven percentage points lower than those observed for reactance.

### 3.3. Correlation Between BIA Parameters and Body Composition

When chemical composition was expressed as a percentage, resistance was negatively correlated with water, protein, ash content, parity, and live weight of rabbit does (*p* < 0.001), as shown in Table 5. Conversely, resistance was positively correlated with fat and energy content, which were also positively associated with each other. Fat and energy content of the rabbit does were positively correlated with live weight (*p* < 0.05) and negatively correlated with parity (*p* < 0.001), water content (*p* < 0.001), ash content (*p* < 0.0001), and protein content (*p* < 0.05). Parity was positively correlated (*p* < 0.05) with live weight, water, protein, and ash contents of the rabbit does. Correlations among variables expressed in absolute values (g) followed the same trend (Table 6).

### 3.4. Regression Equations

The multiple linear regression (MLR) equations developed from the calibration group (CG) are presented in Tables S1–S5, including the estimated coefficients, standard errors (SE), and *p*-Values of each variable, as well as the coefficient of determination (R^2^), residual standard deviation (SD), coefficient of variation (CV), Mallows’ Cp statistic, and model significance (*p*-M) for each equation.

### 3.5. Validation of Prediction Equations

Independent validation results obtained from the multiple linear regression (MLR) equations are summarized in Table 7. Equations predicting chemical composition in grams accounted for a larger proportion of total model variance than those expressed as percentages. Nevertheless, relative mean prediction errors (RMPEs) were comparable across variables, irrespective of expression basis (percentage or grams).

Among the predicted components, fat content showed the greatest variability, with coefficients of variation of 21.5% and 22.0% when expressed as percentages and grams, respectively. Consequently, this variable also exhibited the highest RMPE values (23.9% and 24.6%, respectively) in the independent validation dataset.

For the validation group, paired *t*-tests were performed to compare analyzed and predicted values derived from MLR models. As reported in Table 8, significant differences were detected only for crude protein content (*p* ≤ 0.012), both when expressed as relative and absolute values. The equations slightly underestimated actual protein content by 3.91% and 3.40%, respectively.

The relationship between the analyzed and estimated values obtained from the prediction equations, as well as the distribution of residuals (analyzed-estimated values) associated with each prediction model for each variable, expressed both in relative and absolute values, are shown in Figures S1–S8. When the variables were expressed in grams, the residual distribution was more homogeneous than when expressed as percentages. Figure S1 shows that the residual distribution for body water (%) was homogeneous except for values above 70%, where overestimation occurred. Ash (%) and protein (%) contents were also overestimated for values exceeding 3.4% and 20%, respectively. Conversely, underestimation was observed for ash and protein contents below 3% and 17%, respectively. Fat (%) and energy (kJ/100 g) were underestimated for values below 9% and 800 kJ/100 g (Figure S4), while fat content values above 20% led to overestimation of this variable. The residual distribution for the estimation of water (g), fat (g), and energy (MJ) contents was homogeneous (Figures S6 and S8). However, for values below 110 g of ash and 650 g of protein, there was underestimation of the corresponding variables.

## 4. Discussion

### 4.1. Chemical Composition of Rabbit Does

The weight ranges of the rabbit does used and their chemical composition (expressed both in % and in g) were notably broad and were comparable to those reported in other studies predicting *in vivo* body condition and body chemical composition [17,18]. More recently, Taghouti et al. [2] confirmed strong relationships between body chemical composition and reproductive traits in rabbit does. Changes in rabbit body composition are determined not only by nutritional factors, but also by a range of non-nutritional variables including physiological stage, genotype, reproductive rhythm, reproductive success, and environmental or management conditions [60].

In this study, the digestive tract was included in the chemical analysis because the electrical current passes through the entire body of the live animal, including the gastrointestinal tract. As previously noted [51], water intake was very low during the period in which the BIA measurements were performed (09:00–11:00 a.m.). Therefore, hydration status was assumed to be comparable across all animals, and it was not expected to exert a significant influence on changes in body composition. In fact, the largest variations were associated with shifts in the physiological status of the rabbit does.

The GL females exhibited the highest water content and the lowest proportion of fat and energy. These findings are consistent with earlier reports [3,6] and have been attributed to the high water content of milk and placental fluid. A structural characteristic of this species is the large amount of fluid in the placenta relative to the embryo’s size from very early in gestation. Indeed, the blastocyst at implantation practically occupies the entire lumen of the uterine horn, and by mid-gestation already contains more than 1 mL of fluid [61]. Furthermore, the lower fat and energy proportions in these physiological states are a consequence of the overlap between gestation and lactation, which induces greater mobilization of fat and energy reserves [37,42,43,62].

NGNL rabbit does showed a 6.3% higher protein content than all other experimental groups. This indicates that in GL females, or in females experiencing overlap of gestation and lactation, protein mobilization is greater, necessary for both milk production and fetal growth [6,42,43]. In nulliparous females the lower protein proportion compared to NGNL females likely arises from incomplete growth [2].

### 4.2. Impedance Measurements and Repeatability

The mean resistance values obtained in this work (106 Ω) were higher than those reported in previous studies conducted with pigs or lambs [21,26,28], which ranged between 40 and 50 Ω, despite the latter species having a higher fat content than rabbits. Similarly, in growing animals, resistance values decrease with age, both in growing rabbits (from 120 to 63 Ω between 25 and 77 days of age) [34,35] and in broiler chickens (from 1200 to 185 Ω between 0 and 42 days of age) [36].

These results can be explained by differences in body volume among animals, since impedance values depend on the geometry and volume of the body being measured. Lukaski et al. [22] established that the relationship between body volume and impedance can be expressed as Volume = [(Length)^2^/(Impedance)]^1/2^.

Consequently, larger animals exhibit lower impedance values. Assuming constant geometry and volume across rabbits, the observed differences would then depend solely on body chemical composition, with fatter animals showing higher impedance due to the low conductivity of lipids compared with other body components [24].

The repeatability of bioelectrical impedance analysis (BIA) measurements has been widely studied in humans [63,64,65,66]. The coefficients of variation (CV_R_) for resistance and reactance reported in those studies were lower (between 0.3 and 2.8%, respectively) than those obtained in the present work, which ranged from 10.8% for resistance to 21.6% for reactance. These values were nonetheless lower than those observed in growing rabbits by Saiz et al. [34] (20% and 21.5% for resistance and reactance, respectively). The same authors [35] developed predictive equations based on BIA to estimate rabbit carcass composition, reporting CV_R_ of 15.9% for resistance and 17.6% for reactance. No comparable data have been found for other animal species. A major source of variation may arise beyond the precision of the impedance analyzer itself, from methodological differences between human and animal applications. In humans, electrodes are placed on the skin surface, whereas in rabbit does and young rabbits, the electrical current is applied through subcutaneous needles. The depth of needle insertion, a potential source of variation, could significantly influence the results. These findings therefore support the recommendation that at least two measurements be taken for each rabbit to ensure reliability.

### 4.3. Correlation Between BIA Parameters and Body Composition

The negative correlations observed between resistance and the water, ash, and protein contents of rabbit does, as shown in the correlation matrices where variables are expressed in both % and g (Table 5 and Table 6), can be explained by the fact that fat-free tissues contain a higher proportion of water [21,30]. Consequently, electric current passes more easily through these tissues, a pattern that has also been reported in other species. Parity and live weight of the rabbit does were likewise negatively correlated with resistance, which may reflect the greater body mass and size of older animals; as previously noted, larger body size is associated with lower resistance values [22].

In contrast, resistance was positively correlated with fat and energy contents. Since energy content increases proportionally with fat deposition, these tissues, with their low water content, offer greater resistance to the passage of electrical current [26,30]. A negative correlation was also detected between parity and the fat and energy contents of the rabbit does, which may be attributed to the gradual depletion of body reserves over successive reproductive cycles. Similar trends were reported by other authors [42,43,67], who observed a linear decrease in fat and energy content from the first to subsequent parturitions.

### 4.4. Validation of Prediction Equations

The results of this study indicate that the equations obtained through multiple linear regression (MLR) were robust, as the relative mean prediction error (RMPEs) obtained during independent data validation were not high. Among the variables analyzed, fat content exhibited the highest RMPE (23.9%). However, the estimated fat content in the validation population (13.1%) was very similar to that of the analyzed population (13.2%; *p* = 0.95). When fat was expressed in grams, only minor variations were observed between the estimated and analyzed values (563 *vs.* 567 g), and these differences were not statistically significant.

Because fat and energy composition were highly correlated (r = 0.97; *p* < 0.0001), and the RMPE for energy prediction was lower (15%) than for fat (23.9%), energy values could serve as a more reliable predictor than fat. The differences between analyzed and estimated energy (1004 *vs.* 1021 kJ/100 g, respectively; *p* = 0.57) were comparable in magnitude to those observed for fat. These findings are consistent with those of Fortun-Lamothe et al. [17], who applied the TOBEC method to estimate body composition in breeding does using linear multiple regression. Although these authors did not report RMPE values, they obtained a coefficient of variation for fat prediction close to 25%, slightly higher than that observed in the present study (21.5% and 22% for fat expressed in % and g, respectively). They also found somewhat larger, though non-significant, differences between the calibration and validation datasets compared to those observed here, with overestimations of 3.8% for percentage fat and 4.9% for fat expressed in grams.

In the present study, the only variable showing a significant difference between analyzed and estimated values was protein content (both in relative and absolute values), resulting in an underestimation of 3–4%. Nevertheless, the RMPE for protein (6.50–6.62%; Table 7) was considerably lower than those obtained for fat and energy. In addition, the correlations between analyzed and estimated protein values were higher (r = 0.69 when expressed in %, and r = 0.89 when expressed in g; Figures S1 an S5, respectively) than those obtained for fat (r = 0.51 in % and r = 0.68 in g; Figures S3 and S7, respectively) or energy (r = 0.36 in kJ/100 g and r = 0.74 in MJ; Figures S3 and S7, respectively). These results suggest that the significant differences observed between analyzed and estimated protein values likely stem from the lower intrinsic variability of protein content compared with other chemical components.

The prediction equations estimating the chemical composition of rabbit does expressed in grams yielded a more homogeneous residual distribution, stronger correlations between analyzed and estimated variables, and higher coefficients of determination than equations expressed in percentages. This finding is logical, as the range of variation for the independent variables is narrower when expressed in % than in g, leading to weaker fits. Nevertheless, calibration and prediction errors were similar for each variable regardless of the unit of expression, suggesting that although percentage-based equations exhibit a slightly poorer fit, their predictive accuracy is comparable. Therefore, both types of equations can be used interchangeably, with percentage-based equations offering the additional advantage that variations in body composition are independent of changes in body weight.

Overall, these results align with previous studies [17,42,43,68], which also reported higher total variability explained by models predicting body composition when expressed in grams rather than in percentages.

## 5. Conclusions

It can be concluded that (1) the accuracy in estimating body composition, expressed in both relative and absolute values, was similar, but relative values have the advantage that they do not depend on variations in the body weight of the rabbit does; (2) the bioelectrical impedance analysis (BIA) method can be applied to determine the chemical composition of breeding rabbits during successive reproductive cycles; and (3) BIA accurately predicts the chemical composition of New Zealand × Californian rabbit does, showing values similar to those obtained using the comparative slaughter technique; however, this conclusion applies to the genotype used in the present study, and other genotypes may differ.

## Figures and Tables

**Table 1:** Chemical composition of rabbit does used for calibration and validation.

	Calibration Group (*n* = 87)				Validation Group (*n* = 25)			
Variable	Mean	Minimum	Maximum	SD ^1^	Mean	Minimum	Maximum	SD ^1^
Live weight (g)	4267	3002	5736	533	4260	2837	5014	566
Chemical composition								
Water (%)	61.9	53.0	74.4	4.68	62.9	56.6	70.8	3.42
Ash (%)	3.14	2.58	4.05	0.29	3.23	2.78	3.79	0.25
Protein (%)	17.9	15.7	20.8	0.98	18.6	16.0	21.5	1.40
Lipids (%)	13.7	2.46	23.8	4.65	13.1	6.64	18.4	3.14
Energy (kJ/100 g)	1051	557.3	1372	191	1004	704	1284	138
Water (g)	2638	1787	3632	347	2678	1724	3181	374
Ash (g)	133	99.0	179	18.0	137	97.9	166	17.2
Protein (g)	765	537	1092	101	790	573	987	101
Lipids (g)	595	90.5	1154	234	563	294	908	174
Energy (MJ)	45	21.4	71.2	11.1	42.9	30.3	60.7	9.18

^1^ SD: Standard deviation.

**Table 2:** Body chemical composition of rabbit does by physiological state (Calibration group; *n* = 87).

	Physiological State ^1^						
	PL	PNL	NPL	NPNL	NUL	SEM ^2^	*p*-Value
Number of animals	18	18	18	18	15	−	−
Live weight, g	4490 ^a^	4469 ^a^	4167 ^b^	4310 ^ab^	3748 ^c^	115	<0.001
Relative value, %							
Water	65.4 ^a^	62.7 ^b^	62.3 ^b^	59.6 ^c^	59.4 ^c^	0.36	<0.001
Protein	17.8 ^b^	17.7 ^b^	17.5 ^b^	18.9 ^a^	17.9 ^b^	0.092	<0.001
Lipids	11.1 ^b^	13.5 ^a^	13.7 ^a^	15.1 ^a^	15.8 ^a^	0.39	<0.001
Ash	3.14	3.05	3.17	3.28	3.08	0.034	0.21
Energy (kJ/100 g)	928 ^b^	1044 ^a^	1039 ^a^	1114 ^a^	1147 ^a^	15.1	<0.001
Absolute value, g							
Water	2786 ^a^	2668 ^b^	2652 ^b^	2524 ^c^	2547 ^c^	16.2	<0.001
Protein	758 ^b^	752 ^b^	745 ^b^	808 ^a^	763 ^b^	3.87	<0.001
Lipids	485 ^b^	584 ^ab^	591 ^a^	661 ^a^	665 ^a^	17.2	<0.011
Ash	133	130	135	139	131	1.11	0.23
Energy (MJ)	40.0 ^b^	44.9 ^a^	44.6 ^a^	48.2 ^a^	48.5 ^a^	0.68	<0.001

^1^ PL: pregnant-lactating; PNL: pregnant-non-lactating; NPL: non-pregnant-lactating; NPNL: non-pregnant-non-lactating; NUL: nulliparous. ^2^ SEM: standard error of means. Means within a row without a common superscript differ (*p* < 0.05).

**Table 3:** Mean, minimum, maximum, and standard deviation of bioelectrical impedance analysis (BIA) (*n* = 87) used to develop the prediction equations.

	Mean	Minimum	Maximum	Standard Deviation
*Rs*, Ω	106	67.0	157	20.7
*Xc*, Ω	25.2	11.0	58.0	7.53
D, cm	20.2	16.0	26.0	2.06

*Rs*: resistance; *Xc*: reactance; D: inter-electrode distance.

**Table 4:** Repeatability (S_R_) and coefficient of variation in repeatability (CV_R_) of resistance (*Rs*) and reactance (*Xc*).

	S_R_, Ω	CV_R_, %
*Rs*, Ω	10.8	10.6
*Xc*, Ω	5.22	21.6

**Table 5:** Correlation matrix of resistance (Ω), reactance (Ω), relative values of chemical composition (%), parity order, and live weight (g) in rabbit does *(n* = 87) ^1^.

	*Rs*	*Xc*	Water	Protein	Fat	Ash	Energy	PO	LW
*Rs*	1	0.45 ****	0.40 ****	0.29 ***	0.42 ****	−0.31 ***	0.42 ****	−0.43 ****	−0.29 ***
*Xc*		1	−0.16	−0.05	0.17	−0.19	0.16	−0.21 *	−0.08
Water			1	0.17	−0.96 ****	0.51 ****	−0.99 ****	0.39 ***	−0.23 *
Protein				1	−0.33 **	0.40 ****	−0.26 *	0.23 *	0.12
Fat					1	−0.56 ****	0.97 ****	−0.38 ***	0.29 **
Ash						1	−0.58 ****	0.36 ***	−0.27 *
Energy							1	−0.42 ****	0.25 *
PO								1	0.32 **
LW									1

^1^ *: *p* < 0.05; **: *p* < 0.01; ***: *p* < 0.001; ****: *p* < 0.0001. *Rs:* resistance; *Xc*: reactance; PO: parity order; LW: live weight.

**Table 6:** Correlation matrix of resistance (Ω), reactance (Ω), absolute values of chemical composition (g), parity order, and live weight (g) in rabbit does (*n* = 87) ^1^.

	*Rs*	*Xc*	Water	Protein	Fat	Ash	Energy	PO	LW
***Rs***	1	0.45 ****	−0.51 ****	−0.39 ****	0.27 **	−0.47 ****	0.16	−0.43 ****	−0.29 ****
***Xc***		1	−0.16	−0.10	0.11	−0.20 *	0.07	−0.21 *	−0.08
**Water**			1	0.78 ****	−0.005	0.79 ****	0.15	0.53 ****	−0.28 ***
**Protein**				1	0.39 ****	0.79 ****	0.55 ****	0.40 ****	0.91 ****
**Fat**					1	0.13	0.97 ****	−0.22 *	0.55 ****
**Ash**						1	0.25 *	0.54 ****	0.75 ****
**Energy**							1	−0.15	0.68 ****
**PO**								1	0.32 **
**LW**									1

^1^ *: *p* < 0.05; **: *p* < 0.01; ***: *p* < 0.001; ****: *p* < 0.0001. *Rs:* resistance; *Xc*: reactance; PO: parity order; LW: live weight.

**Table 7:** Prediction equations accuracy assesed with an independent dataset (*n* = 25).

	R^2^	MEC	CV, %	MPE	RMPE, %
Chemical body composition, %					
Water	0.71	2.70	4.36	3.71	5.90
Protein	0.43	0.77	4.31	1.21	6.50
Ash	0.40	0.24	7.52	0.27	8.35
Fat	0.64	2.96	21.5	3.14	23.9
Energy, kJ/100 g DM	0.70	112	10.7	150	14.9
Chemical body composition, g					
Water	0.90	119	4.48	159	5.94
Protein	0.89	35.6	4.65	52.3	6.62
Ash	0.71	10.1	7.57	11.3	8.23
Fat	0.72	131	22.0	139	24.6
Energy, MJ	0.83	4.91	10.9	6.51	15.2

R^2^: determination coefficient; MEC: mean error of calibration; CV: coefficient of variation; MPE: mean prediction error; RMPE: relative mean prediction error.

**Table 8:** Comparison between analyzed and predicted chemical composition (mean [SD]) with multiple linear regression (MLR) using a paired *t*-test.

	Analyzed	Predicted by MLR	*p*-Value
Relative value, %			
Water	62.9 (3.43)	62.7 (3.04)	0.83
Protein	18.6 (1.40)	17.9 (0.79)	0.003
Ash	3.23 (0.26)	3.21 (0.20)	0.62
Fat	13.1 (3.14)	13.2 (3.34)	0.95
Energy, kJ/100 g DM	1004 (138)	1021 (133)	0.57
Absolute value, g			
Water	2678 (374)	2658 (365)	0.54
Protein	790 (101)	764 (86.0)	0.012
Ash	137 (17.2)	136 (15.8)	0.69
Fat	563 (174)	567 (184)	0.88
Energy, MJ	42.9 (9.18)	43.8 (9.11)	0.46

## Data Availability

The data presented in this study are available on request from the corresponding author. The data are not publicly available due to confidentiality requirements.

## Supplementary Materials

The following supporting information can be downloaded at https://www.mdpi.com/article/10.3390/ani15243611/s1, Table S1: Regression coefficients and standard errors determined by multiple linear regression (MLR) for predicting body water content. Table S2: Regression coefficients and standard errors determined by multiple linear regression (MLR) for predicting body protein content. Table S3: Regression coefficients and standard errors determined by multiple linear regression (MLR) for predicting body fat content. Table S4: Regression coefficients and standard errors determined by multiple linear regression (MLR) for predicting body ash content. Table S5: Regression coefficients and standard errors determined by multiple linear regression (MLR) for predicting body energy content. Figure S1: Relationship between estimated and analyzed values of body ash (a), water (b), and protein (c) from multiple linear regression equations, expressed as % (*n* = 87). Figure S2: Residual distribution from the multiple linear regression models for body ash (a), water (b), and protein (c) contents, expressed as % (*n* = 87). Figure S3: Relationship between estimated and analyzed values of body fat (a), and energy (b) from multiple linear regression equations, expressed as % and kJ/100 g (*n* = 87). Figure S4: Residual distribution from the multiple linear regression models for body fat (a) and energy (b) contents, expressed as % and kJ/100 g (*n* = 87). Figure S5: Relationship between estimated and analyzed values of body ash (a), water (b), and protein (c) from multiple linear regression equations, expressed as g (*n* = 87). Figure S6: Residual distribution from the multiple linear regression models for body ash (a), water (b), and protein (c) contents, expressed as g (*n* = 87). Figure S7: Relationship between estimated and analyzed values of body fat (a), and energy (b) from multiple linear regression equations, expressed as g and MJ (*n* = 87). Figure S8: Residual distribution from the multiple linear regression models for body fat (a) and energy (b) contents, expressed as g and MJ (*n* = 87).
