# Supplementary material for: Bioelectrical Impedance Analysis as a Non-Invasive Approach to Estimate In Vivo Body Composition in Rabbit Does Across Physiological Stages
Source: Animals (Basel). 2025 Dec 15;15(24):3611. doi: 10.3390/ani15243611 (PMC12729742; doi:10.3390/ani15243611)
Supplement: Supplementary file 1 [file animals-15-03611-s001.zip › animals-3983657-supplementary.pdf]

Table S1. Regression coefficients and standard errors determined by multiple linear regression (MLR) for predicting body water content.

**Dependent variable**	**Independent variable**	**Estimate**	**SE**	***p*-Value**	**R^2**	**rsd**	**CV (%)**	***p*-M**
**Water, %**	Intercept (NUL)	120.62	10.04	0.0001	0.71	2.70	4.36	0.0001
	Physiological state							
	PL	5.89	1.09	0.0001				
	PNL	4.49	0.99	0.0001				
	NPL	1.79	1.09	0.1086				
	NPNL	−0.63	1.15	0.5838				
	LW^2	-6.42×10^{-7}	7.9×10^{-8}	0.0001				
	PO^2	0.07	0.014	0.0001				
	Rs	−0.69	0.16	0.0001				
	Rs^2	0.0028	7.2×10^{-4}	0.0002				
	Xc	−0.66	0.19	0.001				
	Xc^2	0.0096	0.0027	0.0007				
**Water, g**	Intercept (NUL)	1806.79	729.85	0.0156	0.90	119	4.48	0.0001
	Physiological state							
	PL	222.53	48.26	0.0001				
	PNL	161.24	44.52	0.0005				
	NPL	53.42	48.73	0.2765				
	NPNL	−72.56	51.14	0.1602				
	LW	1.11	0.34	0.0016				
	LW^2	-8.4×10^{-5}	4×10^{-5}	0.0368				
	PO^2	2.93	0.64	0.0001				
	Rs	−34.99	7.33	0.0001				
	Rs^2	0.14	0.032	0.0001				
	Xc	-25.95	8.46	0.0030				
	Xc^2	0.38	0.12	0.0019				

SE: standard error; R^2: determination coefficient; rsd: residual standard deviation; CV: coefficient of variation; *p-M*: model probability.

NUL: Nulliparous; PL: Pregnant-Lactating; PNL: Pregnant-Non Lactating; NPL: Non Pregnant-Lactating; NPNL: Non Pregnant-Non Lactating; Rs: resistance; Xc: reactance; PO: parity order; LW: live weight.

Table S2. Regression coefficients and standard errors determined by multiple linear regression (MLR) for predicting body protein content.

Dependent variable	Independent variable	Estimate	SE	*p*-value	R^2	rsd	CV (%)	*p-M*
**Protein, %**	Intercept (NUL)	20.09	0.720	0.0001	0.43	0.77	4.31	0.0001
	Physiological state							
	PL	0.052	0.31	0.8687				
	PNL	−0.026	0.28	0.3580				
	NPL	−0.07	0.29	0.8148				
	NPNL	1.20	0.31	0.0003				
	LW^2	-7.3×10^{-8}	2.2×10^{-8}	0.0012				
	Rs	0.54	0.23	0.0206				
	Xc	0.17	0.07	0.0130				
	Z	−0.57	0.24	0.0173				
**Protein, g**	Intercept (NUL)	442.93	33.13	0.0001	0.89	35.6	4.65	0.0001
	Physiological state							
	PL	4.47	14.46	0.7581				
	PNL	−5.55	12.78	0.6651				
	NPL	1.66	13.70	0.9036				
	NPNL	55.62	14.42	0.0002				
	LW^2	1.8×10^{-5}	9.9×10^{-7}	0.0001				
	Rs	18.27	10.55	0.0873				
	Xc	5.69	3.05	0.0660				
	Z	−19.30	10.86	0.0795				

SE: standard error; R^2: determination coefficient; rsd: residual standard deviation; CV: coefficient of variation; *p-M*: model probability.

NUL: Nulliparous; PL: Pregnant-Lactating; PNL: Pregnant-Non Lactating; NPL: Non Pregnant-Lactating; NPNL: Non Pregnant-Non Lactating; Rs: resistance; Xc: reactance; LW: live weight; Z: Impedance.

Table S3. Regression coefficients and standard errors determined by multiple linear regression (MLR) for predicting body fat content.

Dependent variable	Independent variable	Estimate	SE	*p-Value*	R^2	rsd	CV (%)	*p*-M
**Fat, %**	Intercept (NUL)	-39.84	11.02	0.0005	0.64	2.96	21.5	0.0001
	Physiological state							
	PL	−4.34	1.19	0.0005				
	PNL	−3.22	1.09	0.0041				
	NPL	−1.24	1.21	0.3060				
	NPNL	0.024	1.27	0.9850				
	LW^2	$6.7x10^{-7}$	$8.7x10^{-8}$	0.0001				
	PO^2	−0.067	0.016	0.0001				
	Rs	0.57	0.18	0.0018				
	Rs^2	−0.0022	$7.9x10^{-4}$	0.0067				
	Xc	0.61	0.21	0.0051				
	Xc^2	−0.0089	0.0029	0.0036				
**Fat, g**	Intercept (NUL)	−2189.89	487.02	0.0001	0.72	131.0	22.0	0.0001
	Physiological state							
	PL	−163.94	52.75	0.0027				
	PNL	−120.06	48.14	0.0148				
	NPL	−40.32	53.32	0.4518				
	NPNL	32.17	55.91	0.5667				
	LW2	$4.4x10^{-5}$	$3.8x10^{-6}$	0.0001				
	PO^2	−2.89	0.69	0.0001				
	Rs	27.67	7.79	0.0007				
	Rs^2	−0.11	0.035	0.0029				
	Xc	25.70	9.31	0.0073				
	Xc^2	−0.38	0.13	0.0046				

SE: standard error; R^2: determination coefficient; rsd: residual standard deviation; CV: coefficient of variation; *p-M*: model probability.

NUL: Nulliparous; PL: Pregnant-Lactating; PNL: Pregnant-Non Lactating; NPL: Non Pregnant-Lactating; NPNL: Non Pregnant-Non Lactating; Rs: resistance; Xc: reactance; PO: parity order; LW: live weight.

Table S4. Regression coefficients and standard errors determined by multiple linear regression (MLR) for predicting body ash content.

Dependent variable	Independent variable	Estimate	SE	*p*-Value	R^2	rsd	CV (%)	*p*-M
**Ash, %**	Intercept (NUL)	4.66	0.30	0.0001	0.40	0.24	7.52	0.0001
	Physiological state							
	PL	0.014	0.096	0.8872				
	PNL	0.043	0.087	0.6257				
	NPL	0.011	0.094	0.9090				
	NPNL	0.16	0.10	0.1251				
	LW	-2.9×10^{-4}	5.6×10^{-5}	0.0001				
	PO	0.035	0.012	0.0034				
	Rs	−0.0040	0.0014	0.0068				
**Ash, g**	Intercept (NUL)	59.91	12.69	0.0001	0.71	10.1	7.57	0.0001
	Physiological state							
	PL	−0.19	4.12	0.9634				
	PNL	1.11	3.73	0.7676				
	NPL	0.40	4.03	0.9207				
	NPNL	6.01	4.43	0.1786				
	LW	0.020	0.0024	0.0001				
	PO	1.50	0.50	0.0037				
	Rs	−0.16	0.06	0.0101				

SE: standard error; R^2: determination coefficient; rsd: residual standard deviation; CV: coefficient of variation; *p-M*: model probability.

NUL: Nulliparous; PL: Pregnant-Lactating; PNL: Pregnant-Non Lactating; NPL: Non Pregnant-Lactating; NPNL: Non Pregnant-Non Lactating; Rs: resistance; PO: parity order; LW: live weight.

Table S5. Regression coefficients and standard errors determined by multiple linear regression (MLR) for predicting body energy content.

Dependent variable	Independent variable	Estimate	SE	*p*-Value	R^2	rsd	CV (%)	*p*-M
**Energy, kJ/100 g**	Intercept (NUL)	−1452.90	416.92	0.0008	0.70	112	10.7	0.0001
	Physiological state							
	PL	−211.77	45.16	0.0001				
	PNL	−158.64	41.21	0.0002				
	NPL	−58.43	45.64	0.2044				
	NPNL	4.10	47.86	0.9320				
	LW^2	$2.8x10^{-5}$	$3.3x10^{-6}$	0.0001				
	PO^2	−3.033	0.59	0.0001				
	Rs	29.11	6.67	0.0001				
	Rs^2	−0.12	0.029	0.0002				
	Xc	27.56	7.97	0.0009				
	Xc^2	−0.40	0.112	0.0006				
**Energy, MJ**	Intercept (NUL)	−94.94	18.28	0.0001	0.83	4.91	10.9	0.0001
	Physiological state							
	PL	−8.08	1.98	0.0001				
	PNL	−5.85	1.81	0.0018				
	NPL	−1.84	2.00	0.3619				
	NPNL	1.65	2.10	0.4346				
	LW^2	$2.4x10^{-6}$	$1.4x10^{-7}$	0.0001				
	PO^2	−0.13	0.03	0.0001				
	Rs	1.42	0.29	0.0001				
	Rs^2	−0.0057	0.0013	0.0001				
	Xc	1.14	0.35	0.0017				
	Xc^2	−0.017	0.0049	0.0011				

SE: standard error; R^2: determination coefficient; rsd: residual standard deviation; CV: coefficient of variation; *p-M*: model probability.

NUL: Nulliparous; PL: Pregnant-Lactating; PNL: Pregnant-Non Lactating; NPL: Non Pregnant-Lactating; NPNL: Non Pregnant-Non Lactating; Rs: resistance; PO: parity order; LW: live weight.

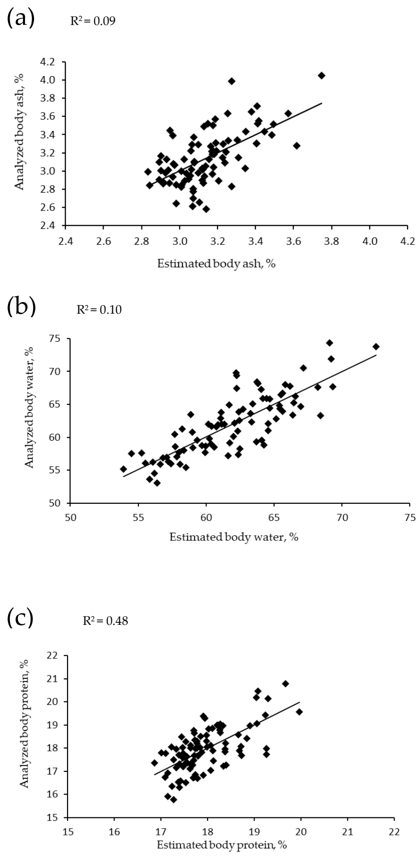

Figure S1. Relationship between estimated and analyzed values of body ash (a), water (b), and protein (c) from multiple linear regression equations, expressed as % (*n* = 87).

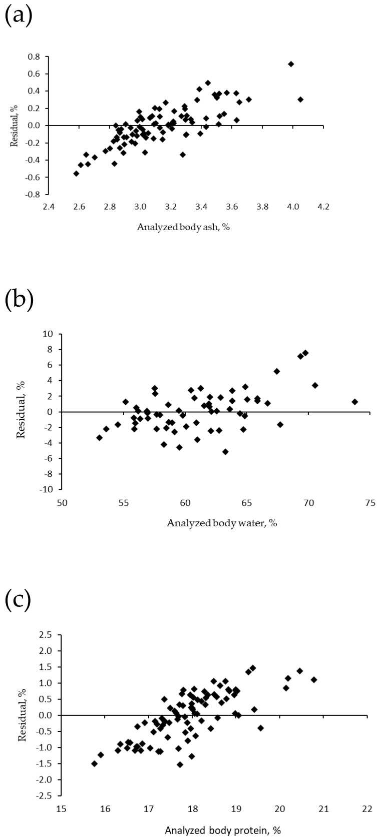

Figure S2. Residual distribution from the multiple linear regression models for body ash (a), water (b), and protein (c) contents, expressed as % (*n* = 87).

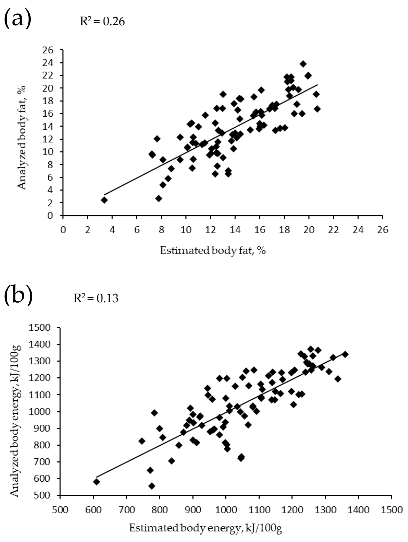

Figure S3. Relationship between estimated and analyzed values of body fat (a), and energy (b) from multiple linear regression equations, expressed as % and kJ/100g (n = 87).

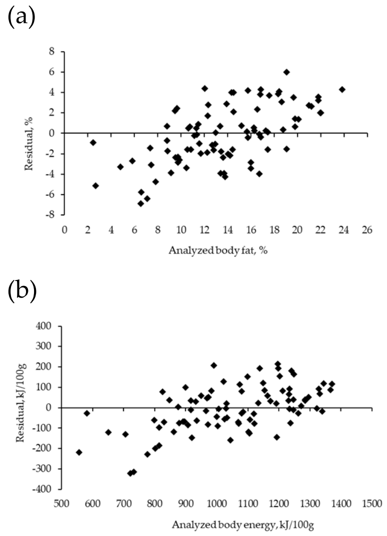

Figure S4. Residual distribution from the multiple linear regression models for body fat (a) and energy (b) contents, expressed as % and kJ/100 g (n = 87).

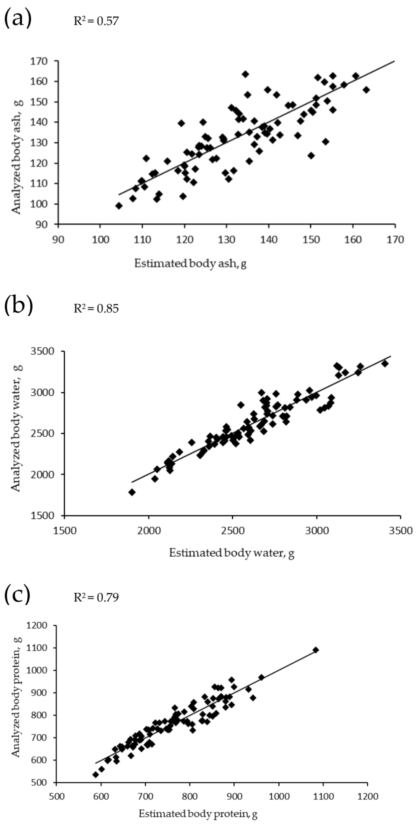

Figure S5. Relationship between estimated and analyzed values of body ash (a), water (b), and protein (c) from multiple linear regression equations, expressed as g *(n* = 87).

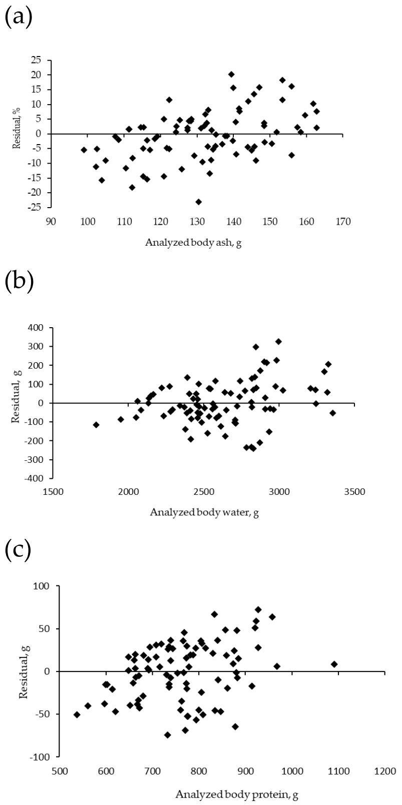

Figure S6. Residual distribution from the multiple linear regression models for body ash (a), water (b), and protein (c) contents, expressed as g (*n* = 87).

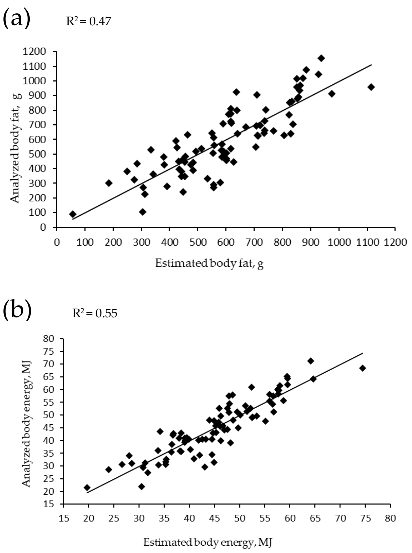

Figure S7. Relationship between estimated and analyzed values of body fat (a), and energy (b) from multiple linear regression equations, expressed as g and MJ (n = 87).

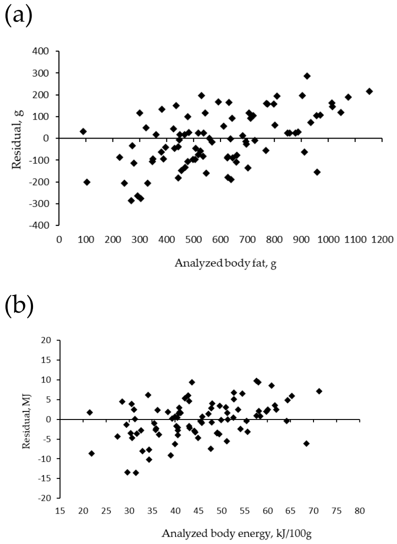

Figure S8. Residual distribution from the multiple linear regression models for body fat (a) and energy (b) contents, expressed as g and MJ (n = 87).
